# Novel compound heterozygous mutations in *WDR62* gene leading to developmental delay and Primary Microcephaly in Saudi Family

**DOI:** 10.12669/pjms.35.3.36

**Published:** 2019

**Authors:** Muhammad Imran Naseer, Mahmood Rasool, Angham Abdulrahman Abdulkareem, Adeel G. Chaudhary, Syed Kashif Zaidi, Mohammad H. Al-Qahtani

**Affiliations:** 1 *Muhammad Imran Naseer, Center of Excellence in Genomic Medicine Research, King Abdulaziz University, 21589, Jeddah, Kingdom of Saudi Arabia. Department of Medical Laboratory Technology, Faculty of Applied Medical Sciences, King Abdulaziz University, 21589, Jeddah, Saudi Arabia*; 2 *Mahmood Rasool, Center of Excellence in Genomic Medicine Research, King Abdulaziz University, 21589, Jeddah, Kingdom of Saudi Arabia. Department of Medical Laboratory Technology, Faculty of Applied Medical Sciences, King Abdulaziz University, 21589, Jeddah, Saudi Arabia*; 3 *Angham Abdulrahman Abdulkareem, Center of Excellence in Genomic Medicine Research, King Abdulaziz University, 21589, Jeddah, Kingdom of Saudi Arabia. Department of Medical Laboratory Technology, Faculty of Applied Medical Sciences, King Abdulaziz University, 21589, Jeddah, Saudi Arabia*; 4 *Adeel G. Chaudhary, Center for Innovation in Personalized Medicine, Faculty of Applied Medical Sciences, Center of Excellence in Genomic Medicine Research, King Abdulaziz University, 21589, Jeddah, Kingdom of Saudi Arabia. Department of Medical Laboratory Technology, Faculty of Applied Medical Sciences, King Abdulaziz University, 21589, Jeddah, Saudi Arabia*; 5 *Syed Kashif Zaidi, Center of Excellence in Genomic Medicine Research, King Abdulaziz University, 21589, Jeddah, Kingdom of Saudi Arabia. Department of Medical Laboratory Technology, Faculty of Applied Medical Sciences, King Abdulaziz University, 21589, Jeddah, Saudi Arabia*; 6 *Mohammad H. Al-Qahtani, Center of Excellence in Genomic Medicine Research, King Abdulaziz University, 21589, Jeddah, Kingdom of Saudi Arabia. Department of Medical Laboratory Technology, Faculty of Applied Medical Sciences, King Abdulaziz University, 21589, Jeddah, Saudi Arabia*

**Keywords:** Compound heterozygous mutation, Primary Microcephaly, Saudi Family, *WDR62*

## Abstract

**Objective:**

Primary microcephaly (MCPH) is a rare autosomal recessive disorder characterized by impaired congenital reduction of brain size along with head circumference and intellectual disability. MCPH is a heterogeneous disorder and more than twenty four genes associated with this disease have been identified so far. The objective of this study was to find out the novel genes or mutations leading to the genetic defect in a Saudi family with primary microcephaly.

**Methods:**

Whole exome sequencing was carried out to find the novel mutation and the results was further validated using Sanger sequencing analysis. This study was done in the Center of excellence in Genomic Medicine and Research, King Abdulaziz University under KACST project during 2017 and 2018.

**Results:**

We report a novel compound heterozygous mutations c.797C>T in exon 7 and c.1102G>A in exon 9 of the WD repeat domain 62 (WDR62) (OMIM 604317) gene in two affected siblings in Saudi family with intellectual disability, speech impediments walking difficulty along with primary microcephaly. Two rare, missense variants were detected in heterozygous state in the *WDR62* gene in these two affected individuals from the heterozygous parents.

**Conclusions:**

A compound heterozygous mutations c.797C>T in exon 7 and c.1102G> A in exon 9 of the *WDR62* gene was identified. *WDR62* gene is very important gene and mutation can lead to neuro developmental defects, brain malformations, reduced brain and head size. These results should be taken into consideration during prognostic discussions and mutation spectrum with affected patients and their families in the Saudi population.

## INTRODUCTION

The autosomal recessive form of primary microcephaly (MCPH) is a rare genetic disorder that is characterized by head circumference less than three standard deviation below the mean from age and sex associated with mild to severe intellectual disability.^1^ Twenty four genes MCPH1-MCPH24 have been reported so far those may be involved in the underlying cause of autosomal recessive primary microcephaly. However, Most of the mutations have been reported in two abnormal spindle microtubule assembly (ASPM) OMIM 608716 genes accounting for more than half of all mutations; and WDR62 gene around 10% of all reported cases related to primary microcephaly.^2^

WD repeat domain 62 genes (WDR62 – GenBank Accession NM_005682.5) are known to play important role in cerebral cortical development and any mutations in this gene lead to cortical malformations, mental retardation and primary microcephaly. Recently a compound heterozygous mutations c.731 C > T (p.Ser 244 Leu) and c.2413 G > T (p.Glu 805 X) in the *WDR62* gene responsible for the mitotic centrosomal protein WDR62, in a microcephaly family from Japanese.^3^ We have also reported in our recent study a missense mutation in exon 30 of WDR62changing alanine to aspartate in the protein leading to the typical MCPH2 phenotype.^4^ Whereas new homozygous splicing variantc.3335+1G>C in the WDR62 gene also reported recently.^5^

Previously pathogenic mutations reported in *WDR62* include missense (e.g. W224S; E526K; R438H) and truncating mutations (e.g. Q470X; Val1402GlyfsTer12; 2083delA; 2472_2473delAG; Gly1280AlafsTer21; c.2527dupG; p.R438H; c.390G>A; p.D955Afs*112).^6^-^12^ In this study two rare, missense variants were detected as compound heterozygous state in the *WDR62* gene of these patients results as c.797C>T, exon 7 (Ala266Val) and c.1102G>A, exon 9 (Asp368Asn) and these mutation leading to the typical MCPH2 MIM 604317 phenotype in Saudi family. The human genome contains two copies of each gene, a paternal and a maternal allele. A mutation affecting only one allele is called heterozygous and affecting both allele called compound heterozygous mutation and if we find any mutation affecting both allele may lead to the disease phenotype. Interestingly, both variants are predicted to be deleterious by the majority of *in silico* prediction tools and are rare in the general population.

## METHODS

### Sample Collections

**Fig.1:**
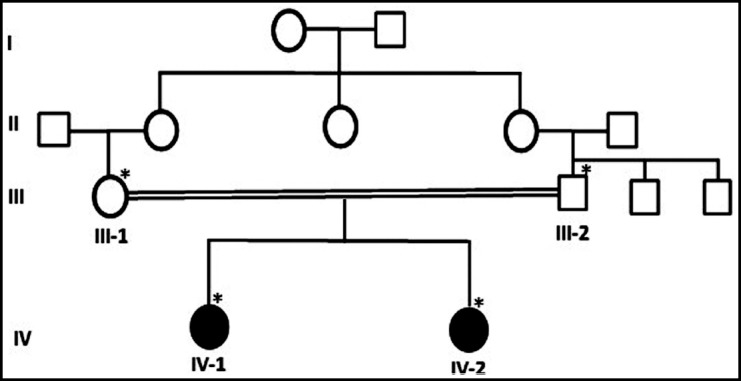
A pedigree of a consanguineous Saudi family representing the primary microcephaly phenotype segregating in an autosomal recessive manner. The samples available for genetic testing are marked with asterisks. Whole exome sequencing was done for IV-1 affected member of the family.

The detailed pedigree (family chart) was drawn after obtaining all the available information from the family as shown in (Fig.1). Detailed written informed consent was taken from all family members and parents before the extraction of blood. This study was approved by the ethical committee of the Center of Excellence in Genomic Medicine Research, King Abdulaziz University Jeddah. The blood samples were collected in the EDTA tube from father, mother and two affected girls. The affected members were under medical examination at Taif Hospital, Saudi Arabia.

### Patient 1 (IV-1)

Patient 1 (IV-1) was 11 years old girl at the time of examination and blood extraction. She presented with proptosis, delayed speech, developmental delay, dysmorphic features and microcephaly. Head circumference was 48 cm<1 percentile -3.8 SD. She also have tumor in the chest cage. She had no other neurological problem such as progressive cognitive decline, seizures and spasticity.

### Patient 2 (IV-2)

Patient 2 (IV-2) was also a 9 years old girl. She had dysmorphic features and developmental delay also has speech problem along with microcephaly. She also has delayed in walking (walking started at the age of 4 years) and was unable to express her feeling. The head circumference was (48 cm <1 percentile (-3.2 SD). She had no other neurological finding, such as seizures, spasticity, or progressive cognitive decline. The phenotype were similar to the previously reported patients associated with *WDR62* gene for primary microcephaly.

### Magnetic resonance imaging (MRI) Examination

**Patient (IV-1):** MRI examination reports multiple bilateral abnormal MR signal foci of viable shape are seen involving the fronto-parieto-occipital region some of them have gyriform pattern. They display high signal in T2 and Fluid-attenuated inversion recovery (FALIR) with restriction in diffusion-weighted imaging (DWIs). The MRI finding rises possibility of ischemic insult. Prominent ventricles and extra axial cerebrospinal fluid (CSF) spaces with no middle shift or deformity was seen. There was no evidence of mass lesion and gross vascular abnormality. Normal cerebellum, brain stem and cervico – medullary junction was seen with normal sellar region.

**Patient (IV-2):** MRI findings report bilateral cortical and subcortical abnormal MR signal patchy area involving occipital region more on right side with mild ischemic insult. They display high signal in T2 and FALIR with faint restriction in DWIs. Normal size shape and position of the ventricles with middle shift or deformity was seen. There was no evidence of mass lesions and gross vascular abnormality. Normal cerebellum, brain stem and cervico – medullary junction were seen with normal sellar region and normal extra axial spaces.

### Whole exome sequencing

Whole exome sequencing was done to identify the pathogenic mutation related to the primary microcephaly. DNA quality and concentration was measured by using 1% agarose gel, 30min running at 100V, 0.5ul of DNA loaded volume. The samples for exome sequencing were prepared according to an Agilent SureSelect Target Enrichment Kit preparation guide (Capture kit, SureSelect V6-Post) and the constructed libraries were prepared and then sequenced using Illumina HiSeq 2000/2500 sequencer. The resulting variant call format (VCF) file contains 107840 variants. These variants were filtered based on frequency, quality, genomic position, protein effect, pathogenicity and based on previous associations with the disease phenotype. We didn’t find any pathogenic variant detected in the known microcephaly genes except we find the compound heterozygous mutation in *WDR62* gene.

### Sanger sequencing

To further confirm the mutation found in whole exome sequencing in the affected members and in the patients Sanger sequencing using Applied Biosystems 3500 (CA, USA) Sequencer (ABI 3500) was performed. To confirm the mutation as pathogenic, we also sequenced this DNA variant in unrelated 100 health control people. *WDR62* gene was amplified by polymerase chain reaction (PCR). PCR products purified and further subjected to cycle sequencing reactions by using BigDye Terminator V3.1 Cycle Sequencing kit to detect any mutation.

In silico analysis and functional prediction of these mutations were analyzed using the available online prediction software tolls that includes, Mutation Taster, PolyPhen-2 (http://genetics.bwh.harvard.edu/pph2/) PROVEAN/SIFT (http://provean.jcvi.org/) and PhastCons (http://compgen.cshl.edu/phast/) etc.

## RESULTS

### Compound Heterozygous Mutations in WDR62 Identified through Exome Sequencing

Whole exome sequencing revealed two rare, missense variants detected in heterozygous state in the *WDR62* gene of this patient. The compound heterozygous missense mutations were in exon 7 and 9 of *WDR62* gene in both affected individuals where c.797C>T, p.(Ala266Val) and c.1102G>A, p.(Asp368Asn).Two rare, missense variants were detected in compound heterozygous state in the *WDR62* gene of two affected patient with primary microcephaly. DNA analysis of parents and affected family members was used to verify cosegregation of the identified variants with the phenotype and establish a compound heterozygous state of these variants. Segregation analysis was done to determine whether these variants are present in compound heterozygous state. Our results showed a compound heterozygous mutation in *WDR62*gene in exon 7 and 9 in the two affected girls. All the mutation for *WDR62* gene known so far is represented in Table-I.

**Table-I:** Mutation spectrum of WDR62 gene mutations known until now.^19^-^23^

S. No	Mutation	Ethnicity	Mutation Type	Alteration	Exon/Intron	Reference
1	c.28G>T	-	Missense	p.Ala10Ser	Exon 1	19
2	c.189G>T	-	Missense	p.Glu63Asp	Exon 2	19
3	c.193 G>A	Arab	Missense	p.Val65Met	Exon 2	8, 14
4	c.332G>C	Pakistani	Missense	p.Arg111Thr	Exon 3	2
5	c.363delT	Mexican	Frameshift	p.Asp112MetfsX5	Exon 4	14
6	c.390G >A	Sudanese	Missense	p.Glu130Glu	Exon 4	12
7	c.535_536insA	Indian	Frameshift	p.Met179fsX21	Exon 5	16
8	c.617 G>C	-	Missense	p.Trp224Ser	Exon 6	7
9	c.731 C > T/c.2413 G > T	Japan	Missense	(p.Ser 244 Leu)/(p.Glu 805 X)	Exon 7/20	3
**10**	**c.797C>T/** **c.1102G>A**	**Saudi**	**Missense**	**p.Ala266Val/p.Asp368Asn**	**Exon7/9**	**Present Study**
11	c.900C>A	Indian	Nonsense	p.Cys300X	Exon 8	16
12	c.1043+1 G>A	Turkish	Splicesite	p.Ser348RfsX63	Intron 8	14
13	c.1143delA	Pakistani	Frameshift	p.H381PfsX48	Exon 9	23
14	c.1194G>A	Pakistani	Missense	p.Trp398	Exon 9	2
15	c.1198G>A	-	Missense	p.E400K	Exon 9	20
16	c.1313G>A/c.2864-2867delACAG	German	Missense/Frameshift	(p.R438H) /(p.D955Afs*112)	Exon 10/22	10
17	c.1313 G>A	-	Missense	p.Arg438His	Exon 10	8
18	c.1408C>T	-	Nonsense	p.Gln470X	Exon 11	7
19	c.1531 G>A	Pakistani	Missense	p.Asp511Asn	Exon 11	8
20	c.1576 G>T	-	Nonsense	p.Glu526X	Exon 12	7
21	c.1576 G>A	-	Missense	p.Glu526Lys	Exon 12	7
22	c.1605_1606InsT	Turkish	Nonsense	p.Glu536X		18
23	c.1821dupT	French Canadian	Frameshift	p.Arg608Serfs*26	Exon 14	21
24	c.1942 C>T	Pakistani	Missense	p.Gln648X	Exon 15	22
25	c.2083delA/c.2472_2473delAAG	-	Frameshift	p.S696fs/p.Q918fs	Exon17/23	9
26	c.2527dupG	Pakistani	Frameshift	p.Asp843GlyfsX3	Exon 21	11
27	c.2867+4_c2867+7delGGTG	Turkish	Frameshift	p.Ser956CysfsX38	Intron 23	14
28	c.3232 G>A	Pakistani	Missense	p.Ala1078Thr	Exon 27	8
29	c.3335+1G>C	Italian	Splicesite	-	-	5
30	c.3361delG	Pakistani	Frameshift	p.Ala1121Glnfs*6	Exon 28	2
31	c.3503G>A	Pakistani	Missense	p.Trp1168*	Exon 29	2
32	c.3839_3855delGCCAAGAGCCTGCCCTG	Pakistani	Frameshift	p.Gly1280AlafsX21	Exon 30	7
33	c.3878C>A	Saudi	Missense	p.Ala1293Asp	Exon 30	4
34	c.3936dupC/3936_3937incC	Caucasian Turkish Pakistani	Frameshift	p.Val1314ArgfsX18/Val1314GlyfsX17	Exon 30	8, 14
35	c.4205delTGCC	Turkish	Frameshift	p.Val1402GlyfsX12	Exon 31	7
36	c.4241dupT	Pakistani	Frameshift	p.Leu1414LeufsX41	Exon 31	8

### Sanger sequencing

**Fig.2:**
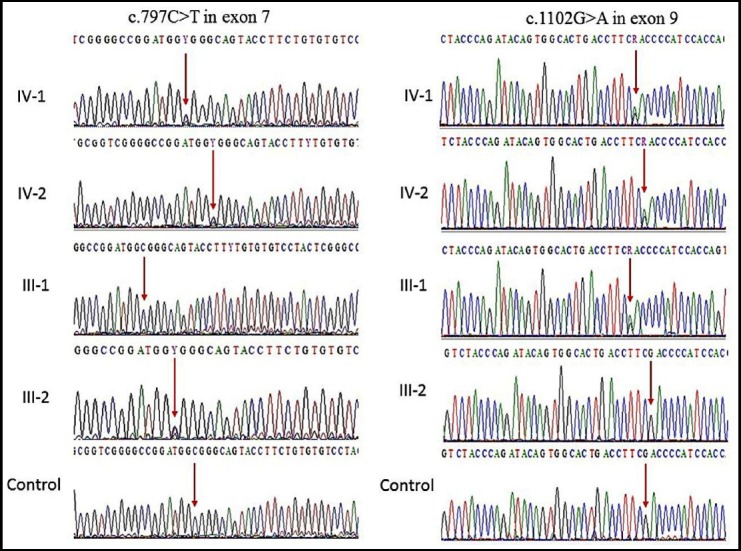
Sanger sequence analysis showed a compound heterozygous mutation in WDR62 gene where (c.982G>A) and (c.1273T>A) in exon 8 of the both affected IV-1, and IV-2 proband whereas the one parent was heterozygous at one position while other was heterozygous at other position.

Our Sanger sequencing results showed a compound heterozygous mutation in *MCPH1* gene where at 982 (c.982G>A) and at position 1273 (c.1273T>A) in exon 8 of the both affected IV-1, and IV-2 proband whereas the one parent was heterozygous at one position while other was heterozygous at other position as shown in (Fig.2). The found mutation was further validated in 100 control samples, but no one has this sequence variation. Both the parents of the affected members were heterozygous at different positions, which also confirm the compound heterozygosity in this family.

### In silico Analysis

The detailed bioinformatics and functional prediction of identified mutations for deleterious effects were completed using the online *In silico* prediction software package PROVEAN/SIFT, PolyPhen-2, and PhastCons as mentioned in Table-II. Furthermore, in the Greater Middle East (GME) minor allele frequency was 0.00 in the database. Further, SIFT 0.12, PolyPhen 0.7, PhyloP (phyloP46way_placental) MutationAssessor 2.0 (0.9) and Mutation Tester (-0.99) predicted this variation as disease causing mutation. This mutations was not reported in the Human Gene Mutation database (HGMD, www.hgmd.cf.ac.uk/) and Online Mendelian Inheritance in Man (MIM/OMIM). 1000 genome (http://www.internationalgenome.org/) and The Exome Aggregation Consortium (ExAc) (Version 0.3.1) (http://exac.broadinstitute.org/) data base. All of the software’s predicted this mutation to be disease causing and lethal for overall proper functioning of the protein as shown in Table-II.

**Table-II:** Shows the results of in silico analysis tools used for prediction of pathogenicity of mutation.

S. No	Online Tools	Release/updated Date	Pathogenicity Score for position 797C>T, exon 7	Pathogenicity Score for position 1102G>A, exon 9
1	SIFT	Updated on 25 July 2017	0.01	0.01
2	1000 Genomes	2013-05-02	0.0	0.0
3	Exome Aggregation Consortium Version 0.3.1	March 14, 2016	0.0	0.0
4	Polyphen-2 (v2.2.2, released in Feb, 2013)	May 30, 2012	0.62	0.68
5	MutationTaster 2	2.0	1.0	1.0
6	MutationAssessor 2.0	Release 2.0	1.23	2.22
7	PhyloP (phyloP46way_placental)	Feb. 2009	0.94	1.05
8	Phastcons 1.4	October 2016	0.99	1.0
9	SiPhy 0.5	May 1, 2009	18.07	18.27

## DISCUSSION

*WDR62* encodes a protein which is required for cerebral cortical development and neurogenesis.^13^ It is proposed to play a role in neuronal migration and proliferation.^7^,^14^ The Wdr62 expression was found to developing mouse brain, with maximum expression in the forebrain.^14^ Wdr62 gene genetically interacts with Aurora A to regulate mitotic progression, spindle formation and maintaining the size of brain. Whereas loss of these gene interactions leads to delay in mitosis and cell death of neural progenitor cells (NPCs) which may cause of human primary microcephaly.^15^

Mutations in *WDR62* have been associated with primary microcephaly 2 (MCPH2), with or without cortical malformations.^14^,^16^,^17^ This is a disease characterized by microcephaly associated with other manifestation and showing a wide phenotypic variability.^18^ Associated features include modest to severe mental retardation, and numerous type of cortical malformations in patients with primary microcephaly. Cortical malformations may include cortical thickening, pachygyria lissencephaly, microgyria, schizencephalyhypoplasia of the corpus callosum. Most of the affected individuals have delayed psychomotor development and having seizures in many cases. Based on the referral note for this patient, mutations in *WDR62* could be of relevance for the reported microcephaly. Additional clinical evaluation and investigations (EEG, MRI) are needed to determine the relevance of this variant. The here detected variants cause two alterations of conserved residues (p.Asp368Asn and p.Ala288Val). p.Asp368Asn alters a residue within the WD5 domain, whereas p.Ala288Val is located in between WD3 and WD4. The main function of all WD-repeat proteins is to coordinate multi-protein complex assemblies, whereas for the protein interactions these repeating units serve as a rigid scaffold. Both detected mutations have not been previously reported.

## CONCLUSION

We have identified a novel compound heterozygous c.797C>T in exon 7 and c.1102G>A mutations in exon 9 of the *WDR62* gene in two affected siblings of Saudi family with intellectual disability, speech impediments walking difficulty and primary microcephaly. We suggest that these type of studies are required to identify complete mutation spectrum related to primary microcephaly which will be useful for the precise clinical diagnosis of individuals suffering from disease in Saudi population.
